# Lysophosphatidic acid accelerates lung fibrosis by inducing differentiation of mesenchymal stem cells into myofibroblasts

**DOI:** 10.1111/jcmm.12178

**Published:** 2013-11-19

**Authors:** Na Tang, Yanxia Zhao, Ruopeng Feng, Yinan Liu, Shuling Wang, Wanguo Wei, Qiang Ding, Michael (Songzhu) An, Jinhua Wen, Lingsong Li

**Affiliations:** a Peking University Stem Cell Research Center Department of Cell Biology School of Basic Medical Sciences, Peking University Health Science CenterBeijing, China; b SARI Center for Stem Cell and NanoMedicine Shanghai Advanced Research Institute, China Academy of SciencesShanghai, China; c Curegenix Inc.Guangzhou, China

**Keywords:** mesenchymal stem cells, lysophosphatidic acid, lung fibrosis, myofibroblasts, Antalpa1

## Abstract

Lung fibrosis is characterized by vascular leakage and myofibroblast recruitment, and both phenomena are mediated by lysophosphatidic acid (LPA) *via* its type-1 receptor (LPA1). Following lung damage, the accumulated myofibroblasts activate and secrete excessive extracellular matrix (ECM), and form fibrotic foci. Studies have shown that bone marrow-derived cells are an important source of myofibroblasts in the fibrotic organ. However, the type of cells in the bone marrow contributing predominantly to the myofibroblasts and the involvement of LPA-LPA1 signalling in this is yet unclear. Using a bleomycin-induced mouse lung-fibrosis model with an enhanced green fluorescent protein (EGFP) transgenic mouse bone marrow replacement, we first demonstrated that bone marrow derived-mesenchymal stem cells (BMSCs) migrated markedly to the bleomycin-injured lung. The migrated BMSC contributed significantly to α-smooth muscle actin (α-SMA)-positive myofibroblasts. By transplantation of GFP-labelled human BMSC (hBMSC) or EGFP transgenic mouse BMSC (mBMSC), we further showed that BMSC might be involved in lung fibrosis in severe combined immune deficiency (SCID)/Beige mice induced by bleomycin. In addition, using quantitative-RT-PCR, western blot, Sircol collagen assay and migration assay, we determined the underlying mechanism was LPA-induced BMSC differentiation into myofibroblast and the secretion of ECM *via*LPA1. By employing a novel LPA1 antagonist, Antalpa1, we then showed that Antalpa1 could attenuate lung fibrosis by inhibiting both BMSC differentiation into myofibroblast and the secretion of ECM. Collectively, the above findings not only further validate LPA1 as a drug target in the treatment of pulmonary fibrosis but also elucidate a novel pathway in which BMSCs contribute to the pathologic process.

## Introduction

Lung fibrosis is a common basic pathological process in many chronic respiratory diseases. It has two main characteristics: vascular leakage and the accumulation of myofibroblasts that secrete excess extracellular matrix (ECM) such as collagen 1–4. Therefore, addressing the question of which types of cells become myofibroblasts will provide insight into the cellular and molecular basis of the disease. Traditionally, it is thought that myofibroblasts are mainly derived from activated lung interstitial fibroblasts 5–6. Upon lung injury, non-contractile fibroblasts convert into activated myofibroblasts that express α-smooth muscle actin (α-SMA). The transition of epithelial cells to fibroblasts is an additional origin for myofibroblasts 7–10. However, there is also evidence to suggest that some of the myofibroblasts in the damaged lung are actually from bone marrow 11–14. In irradiated mice, for example, bone marrow cells contributed significantly to myofibroblasts 12, and accordingly, inhibition of myofibroblast emigration to the damaged lung attenuated the fibrosis 13,14. Nonetheless, many reports present contradictory observations. It has been noted that early intravenous administration of bone marrow stromal cells could ameliorate the fibrotic injuries in mice 16. In addition, recent clinical trials have used bone marrow-derived stromal cells for treating human lung disorders 17–18. It is therefore very crucial to know which types of bone marrow cells become myofibroblasts during lung fibrosis.

Bone marrow cells contain two major subpopulations which are capable of becoming myofibroblasts and appear to be involved in lung fibrosis: mesenchymal stem cells (MSCs) 19,20 and fibrocytes, with the latter also known as bone marrow-derived mesenchymal progenitors 22–28. Fibrocytes are derived from precursors of the monocyte lineage 25–29. Unlike MSCs that express CD44 and Sca-1, fibrocytes are positive for CD45 and collagen I antigens. It has been noted that 80% of collagen I-expressing cells at the fibrotic lesions were of bone marrow origin in lung fibrosis 30–31. However, CD45-positive fibrocyte-derived myofibroblasts accounted for only a portion of the total myofibroblasts 29, implying that a percentage of myofibroblasts may come from bone marrow-derived mesenchymal stem cells (BMSCs) in lung fibrosis.

Lysophosphatidic acid (LPA) is a multi-functional phospholipid which regulates many physiological and pathological activities *via* its type 1- 6 receptors (LPA1- 6) 32. In normal conditions, it takes part in kinds of physiological activities such as neural system development 33 and cardiovascular formation 34. In abnormal conditions, it participates in many pathological diseases such as injury repair 35, neuropathic pain 36, cancer cells invasion 37 and organ fibrosis 4. In particular, the LPA-LPA1 signalling has been reported to be involved in pulmonary fibrosis by mediating resident fibroblast accumulation and vascular leakage 3–4. It also noted that LPA-LPA1 signalling could regulate migration 38, apoptosis 39 and differentiation 40 of MSCs. Nonetheless, the LPA-LPA1 signal-mediated differentiation of MSCs in pulmonary disease has so far not yet been documented.

In this study, we show that BMSCs are an important source of myofibroblasts in the fibrotic lung and that the underlying mechanism is BMSC differentiation into myofibroblast *via* the LPA-LPA1 signalling pathway. In addition, we provide evidence that the novel LPA1 antagonist Antalpa1 attenuates lung fibrosis by inhibiting BMSC differentiation and ECM secretion. These results suggest that Antalpa1 could be a potential clinical drug for fibrotic disease.

## Materials and methods

### Mice and treatment

Bone marrow from ICR mice (6–8 weeks) was replaced with that from EGFP 51 transgenic mice as previously described 41. The mice then received intratracheal administrations of bleomycin (BLM; Melonepharma, Dalian, China) 10 mg/kg bodyweight dissolved in 100 μl saline, to induce lung fibrosis. Mice were injected subcutaneously with Antalpa1 (20 mg/kg/day) or with the same volume of vehicle. In the severe combined immune deficiency (SCID)/Beige mouse lung injury model, we first intratracheally injected BLM in 8-week-old SCID/Beige mice (Vital River, Beijing, China), and then transplanted GFP-labelled hBMSCs (2.0 × 10^6^) or EGFP transgenic mBMSCs *via* caudal vein 48 hrs after BLM administration. The BLM-treated mice were then injected subcutaneously with Antalpa1 or with the same volume of vehicle. Antalpa1 injections began 1 day prior to BLM administration and were repeated daily for 14 days. Mice lung tissues were isolated at 0, 3 or 4, 7, 10, and 14 days after thorough perfusion under deep anaesthesia. All research involving animals was approved by the Peking University Animal Ethics Committee and all efforts were made to minimize suffering.

### mBMSC isolation and characterization

Bone marrow aspirates were obtained from the femur and tibia of 6- to 8-week-old EGFP transgenic mice after deep anaesthesia. Mouse BMSCs were isolated, cultured and characterized as previously reported 41. Briefly, bone marrow aspirates were flushed with α-MEM (Gibco, Grand Island, NY, USA) containing 20% FBS (Gibco, Grand Island, NY, USA) and 1% penicillin–streptomycin. The cell solution was gently beaten to make it a single-cell suspension, plated it on a 100mm dish and then cultured it at 37°C, 5% CO_2_. Twenty-four hours later, cells were changed to new culture medium after washing the cells twice with 1× PBS gently. Mouse BMSCs were passaged for three times and characterized by flow cytometry analysis before collection for use. Antibodies used for flow cytometry are listed in Table S1.

### hBMSC culture and treatment

Commercially available hBMSCs were purchased from Cyagen (Guangzhou, China) and maintained as adherent cultures in Complete Mesenchymal Stem Cell Growth Medium (HUXMF-90011; Cyagen) at 37°C and 5% CO_2_. The culture medium was changed every 2 days, and the cells were split when they reached 80–90% confluence. The cells were used at early passage (<5 passages) for all experiments. For *in vivo* transplantation, cells were turned GFP positive by adenoviral (without gene target) infection. The manufacturer had identified the cells by flow cytometry and differentiation ability analysis.

### Migration assay

Cell migration was determined in Boyden chambers as described previously 41. Briefly, serum-starved hBMSCs or mBMSCs were pre-treated for 0.5 hr with or without Ki16425 (Selleck, Houston, TX, USA) and Antalpa1. The cells (1.0 × 10^5^) were then seeded to the upper chamber (Corning, New York, NY, USA). Cell migration was allowed to proceed for 16 hrs at 37°C in 5% CO_2_ by adding 10 μM LPA (Sigma-Aldrich, St. Louis, MO, USA) to the lower chamber. Cells that migrated to the lower surface of the filter were fixed with 4% PFA and then stained with haematoxylin and eosin. Twenty pictures were taken randomly by ordinary microscope (Olympus, BX51, Tokyo, Japan) and the total number of migrated cells was quantified by cell counting.

### Histology analysis

Lung tissues were formalin-fixed, dehydrated and then embedded in paraffin or O.C.T. (Sakura, CA, USA). Haematoxylin and eosin-staining, immunohistochemical staining and Masson Trichrome staining were performed on paraffin sections. Frozen sections and fixed cell slides were immunofluorecently stained as previously described 42 and the antibodies used are listed in Table S1. For quantitative analysis, 10 photographs were taken randomly with a laser scanning confocal microscope (Leica, SP5, Wetzlar, Germany) or ordinary fluorescence microscope (Olympus, BX51, Tokyo, Japan) per immunofluorecently-stained lung section, 10 sections per mouse and six mice per group.

### Western blot

Total protein extraction and western blot analysis were performed as previously described 42. Briefly, lung tissue or cell proteins were resolved by 10% SDS-PAGE and blotted onto nitrocellulose filter membranes. Membranes were incubated with primary antibodies overnight at 4°C after blocking with 5% non-fat milk for 1 hr. Membranes were then incubated with secondary antibodies for 1 hr at room temperature. Protein bands were visualized by the Odyssey system (LI-COR Bioscience, Lincoln, NE, USA). The antibodies used for western blot are listed in Table S1.

### Quantitative RT-PCR

Total RNA isolation, cDNA reverse transcription and quantitative RT-PCR were performed as previously described 42. Briefly, total RNA was extracted from lung tissues or cells using the RNeasy kit (Qiagen, Dusseldorf, Germany) and reverse transcribed into cDNA according to the manufacturer’s instruction. Quantification of the selected genes by quantitative RT-PCR was performed in an MX7300 sequence detecting system. Sequences of the primers are given in Table S2.

### Sircol collagen assay

The Sircol collagen assay (Biocolor, Northern Ireland, UK) was performed following the manufacturer’s instructions. Samples were from lung tissues and cultured cells.

### ELISA

Commercially available ELISA kits were used to evaluate the levels of LPA, S1P (Echelon, Salt Lake city, UT, USA), and TGF-β1 (R&D system, Minneapolis, MN, USA) in lung tissue and plasma.

### Apoptosis analysis

Human bronchial epithelial cells 16HBE (Bogoo, Shanghai, China) were seeded in ultra low attachment dishes and the cells were cultured in RPMI1640 with 10% FBS and 1% P/S. Twenty-four hours later, cells were pre-treated with or without 30 μM Antalpa1 and were changed to the culture medium that contained 10 μM LPA. Seventy-two hours later, the apoptotic cells were assessed by Annexin V- FITC detection Kit (Baosai, Beijing, China). In brief, 1–5 × 10^5^ cells were collected, washed and resuspended in 200 μl binding buffer. Ten microlitres of AnnexinV-FITC was added to the cell suspension and incubated in the dark at room temperature for 15 min after gentle mixing. Three hundred microlitres of binding buffer and 5 μl of PI were added to the tube. Flow cytometry (FACS Calibur, BD, Franklin Lakes, NJ, USA) was performed at an excitation wavelength of 488 nm and emission wavelength of 530 nm.

### Statistical analysis

Results were analysed by Student’s *t*-test. Differences were considered statistically significant if *P* values were less than 0.05.

## Results

### Myofibroblasts in bleomycin-induced fibrotic lung may be derived from BMSCs

**Figure 1:**
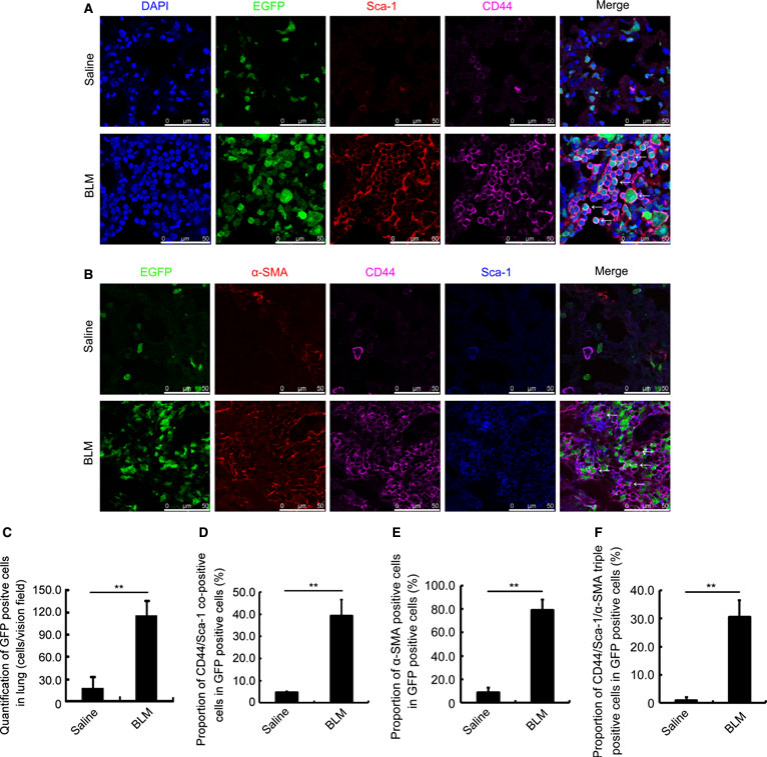
Myofibroblasts in bleomycin-damaged lung could be derived from bone marrow derived-mesenchymal stem cells. (A) Immunofluorescence staining for EGFP (green), Sca-1 (red), and CD44 (purple) in the lung of Saline- (upper) or BLM-(lower) challenged mice with EGFP bone marrow replacement. DAPI (blue) staining shows the nucleus. EGFP/Sca-1/CD44 co-positive cells are indicated with arrows. (B) Immunofluorescence staining for Sca-1 (blue), CD44 (purple), and α-SMA (red) in the lung of Saline- (upper) or BLM-(lower) challenged mice with EGFP bone marrow replacement. EGFP/Sca-1/CD44/α-SMA co-positive cells are indicated with arrows. (C) Quantification of total EGFP-positive cells in the lung of Saline and BLM groups with EGFP transgenic bone marrow replacement. (D) Proportion of EGFP/Sca-1/CD44 co-positive cells in the lung of Saline and BLM groups with EGFP transgenic bone marrow replacement. (E) Proportion of EGFP/α-SMA co-positive cells in the lung of Saline and BLM groups with EGFP transgenic bone marrow replacement. (F) Proportion of EGFP/CD44/Sca-1/α-SMA co-positive cells in the lung of Saline and BLM groups with EGFP transgenic bone marrow replacement. Each value represents the mean ± SE. *n* = 6 mice in each group. **P < 0.01. BLM: bleomycin.

Administration of BLM in mice whose bone marrow has been replaced by that of EGFP transgenic ‘green’ mice caused lung fibrosis in all treated animals as expected (Fig. S1A). In the fibrotic lung, the total number of ‘green cells’ derived from bone marrow increased approximately eightfold (Fig. 1A and C, BLM). Along with an increase in cell number, the ratio of CD44/Sca-1-positive BMSCs among the ‘green’ cells increased approximately eightfold from less than 5% (Fig. 1A and D, Saline) to 40% in the damaged lung (Fig. 1A and D, BLM). However, few CD44/Sca-1/EGFP-positive cells co-stained with CD45 in the thoroughly perfused lung of mice challenged with or without BLM (Fig. S1B, Merge). From these results, we show that MSCs other than haematopoietic progenitor cells derived from bone marrow largely migrate into the damaged lung.

To determine the composition of cells accumulated in the fibrotic region, lung tissue from mice challenged by BLM were stained with anti-α-SMA, anti-CD44 and anti-Sca-1 antibodies. We found that nearly 80% ‘green’ cells were α-SMA positive and more than 30% ‘green’ cells were CD44/Sca-1/α-SMA triple positive in the BLM challenged mice (Fig. 1B, E and F, BLM). Compared to that in saline-challenged mice (Fig. 1B, E and F, Saline), this triple-positive subpopulation increased ∼30-fold after bleomycin treatment. Furthermore, ∼40% α-SMA-positive cells were ‘CD44/Sca-1-positive green cells’ (Fig. S4A, BLM). However, less than 30% α-SMA-positive cells were derived from CD45-positive ‘green’ cells (Fig. S4B) and nearly 10% α-SMA-positive cells were from fibroblast-specific protein-1 (FSP1)-positive ‘green’ cells (Fig. S4C). These suggest that the largest proportion of α-SMA-positive cells was from BMSCs and BMSCs are one of the major cell sources contributing to myofibroblasts in the fibrotic lung.

### Significant increase in LPA concentration in the fibrotic lung

**Figure 2:**
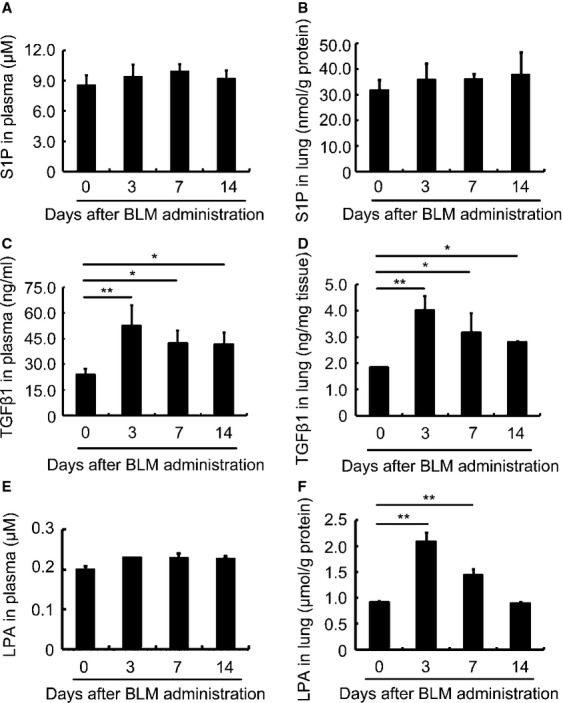
Lysophosphatidic acid (LPA) concentration increases in the fibrotic lung. (A) S1P concentration in the plasma of mice 0, 3, 7 and 14 days after BLM administration detected by ELISA. (B) S1P concentration in lung tissue of mice 0, 3, 7 and 14 days after bleomycin administration detected by ELISA. (C) TGF-β1 concentration in the plasma of mice 0, 3, 7 and 14 days after BLM administration detected by ELISA. (D) TGF-β1 concentration in lung tissue of mice 0, 3, 7 and 14 days after BLM administration detected by ELISA. (E) LPA concentration in the plasma of mice 0, 3, 7 and 14 days after BLM administration detected by ELISA. (F) LPA concentration in lung tissue of mice 0, 3, 7 and 14 days after BLM administration detected by ELISA. Each value represents the mean ± SE. *n* = 5 mice in each group. *P < 0.05; **P < 0.01. BLM: bleomycin.

As LPA-LPA1 signalling, together with TGF-β1, plays an important role in the accumulation of myofibroblasts 3–44, we measured both LPA and TGF-β1 levels in the lungs of mice treated with BLM. As a control, we also measured the sphingosine-1-phosphate (S1P) concentration in the damaged lung, as our laboratory has previously demonstrated that S1P regulates BMSC migration in chemically damaged mouse liver 41. As shown, S1P levels did not change in the plasma (Fig. 2A) or in the damaged lung (Fig. 2B). However, TGF-β1 increased in both the plasma (Fig. 2C) and the damaged lung (Fig. 2D) isolated from mice at days 0, 3, 7 and 14 upon BLM administration, suggesting that an increase in TGF-β1 is a systematic response to lung damage. LPA concentration did not change in the plasma (Fig. 2E) but increased significantly only in the lung (Fig. 2F), suggesting that the increase in LPA was from the primary damaged site rather than from the peripheral circulation. The increase in LPA lasted a week and declined to the basal level at day 14 (Fig. 2F).

### A novel LPA1 selective antagonist, Antalpa1

**Figure 3:**
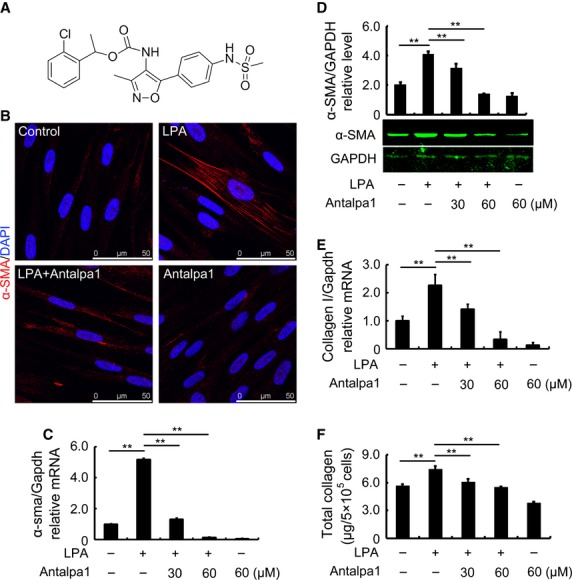
Antalpa1 inhibits hBMSC differentiation into myofibroblasts induced by lysophosphatidic acid (LPA) *in vitro*. (A) Chemical structure of Antalpa1 (CGX-1002). (B) Immunofluorescence staining for α-SMA (red) in hBMSCs with or without stimulation. DAPI (blue) staining shows the nucleus. The images stand for without addition (Control), 10 μM LPA (LPA), 10 μM LPA with 30 μM Antalpa1 pre-treatment (LPA + Antalpa1) and 30 μM Antalpa1 pre-treatment (Antalpa1) respectively. (C) Relative mRNA expression of *α-sma* in hBMSCs with or without stimulation detected by quantitative RT-PCR analysis. (D) Protein levels of α-SMA in hBMSCs with or without stimulation detected by western blot analysis. (E) Relative mRNA expression of collagen I in hBMSCs with or without stimulation detected by quantitative RT-PCR analysis. (F) Total protein levels of collagen in hBMSCs with or without stimulation detected by Sircol collagen assay. Each value represents the mean ± SE. *P < 0.05; **P < 0.01. The experiment was repeated more than three times.

To investigate the role of LPA-LPA1 signalling in the regulation of MSCs in animals with lung fibrosis, we used a robust high-throughput *in vitro* assay to screen a diverse small molecule library for modulators of LPA1. One of the confirmed hits from this screen was Antalpa1 (Fig. 3A, antagonist CGX-1002, IC_50_ = 7.2 μM for LPA1). Antalpa1 was chemically synthesized according to supplemental product information. The antagonism for LPA1 receptor and its selectivity for other LPA receptors were determined by a serum response element (SRE) reporter gene assay and a Ca^2+^ influx assay using cells expressing human LPA1–5 receptors. Antapla1 inhibited LPA-induced SRE activation and the intracellular release of calcium from 293 cells transiently expressing human LPA1, and showed no effect on other LPA receptors at the same concentration range (Table S3). It is likely that Antalpa1 surpasses other published LPA1 antagonists such as Ki16425 and will be a good candidate particularly for *in vivo* studies.

### LPA-LPA1 signalling induces differentiation of hBMSCs into myofibroblasts

To investigate the function of LPA-LPA1 signalling, we first measured the effect of LPA-LPA1 signalling on hBMSC migration using a Boyden chamber assay. As shown, LPA indeed induced hBMSC migration (Fig. S2A and B, LPA) and Ki16425, an antagonist for both LPA1 and LPA3 4, completely blocked this LPA effect (Fig. S2A and B, LPA+Ki16425). However, the novel LPA1-specific inhibitor, Antalpa1 could block some hBMSC migration, but not significantly (Fig. S2A and B, LPA+Antalpa1). These data suggest that it is mainly LPA3, but not LPA1 that mediates the function of LPA in the regulation of hBMSC migration.

Therefore, we further investigated the effect of LPA-LPA1 signalling on the differentiation of hBMSCs into myofibroblasts. After 48 hr incubation with LPA, most cells became positive for α-SMA expression (Fig. 3B, LPA). Accordingly, both mRNA expression (Fig. 3C) and protein production of α-SMA (Fig. 3D) were significantly elevated after the induction. This increase disappeared if the cells were pre-treated with Antalpa1 (Fig. 3C and D). It appears that 30 μM Antalpa1 could completely block the effect of LPA on the differentiation of MSCs into myofibroblasts (Fig. 3B, LPA+Antalpa1 and Fig. 3C and D).This was also the case for collagen I mRNA and protein (Fig. 3E and F). We therefore show that LPA-LPA1 signalling stimulates hBMSC differentiation into myofibroblast and the secretion of ECM.

### Antalpa1 attenuates lung fibrosis by inhibiting BMSC differentiation into myofibroblast

**Figure 4:**
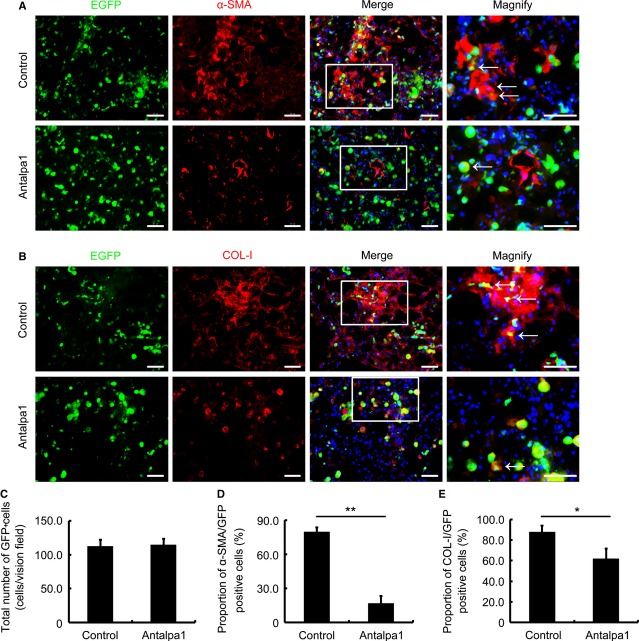
Antalpa1 reduces BM-derived cell differentiation into myofibroblasts and the secretion of extracellular matrix in bleomycin-damaged lung. (A) Immunofluorescence staining for α-SMA (red) in the lung of bleomycin-(BLM) challenged mice (EGFP bone marrow-replaced) with Control (upper) or Antalpa1 (lower) injection. DAPI (blue) staining shows the nucleus. α-SMA/GFP co-positive cells are indicated with arrows. Bar = 30 μm. (B) Immunofluorescence staining for collagen I (COL-I, red) in the lung of BLM-challenged mice (EGFP bone marrow-replaced) with Control (upper) or Antalpa1 (lower) injection. DAPI (blue) staining shows the nucleus. COL-I/GFP co-positive cells are indicated with arrows. Bar = 30 μm. (C) Quantification of GFP-positive cells in the lung of Control and Antalpa1 groups. (D) Proportion of α-SMA/GFP co-positive cells in the lung of Control and Antalpa1 groups. (E) Proportion of COL-I/GFP co-positive cells in the lung of Control and Antalpa1 groups. Each value represents the mean ± SE. *n* = 6 mice in each group. *P < 0.05; **P < 0.01.

To test our hypothesis *in vivo*, we first examined the effect of Antalpa1 on the migration and differentiation of bone marrow-derived ‘green’ cells in the lung of BLM-treated mice (EGFP transgenic bone marrow replaced). As compared with that in the lung of control animals (Fig. 4 A–C, Control), the total number of ‘green’ cells remained unchanged in the lungs of Antanlpa1-treated animals (Fig. 4 A–C, Antalpa1). However, the proportion of α-SMA/GFP co-positive cells reduced from ∼80% in the control (Fig. 4A and D, Control) to ∼15% (Fig. 4A and D, Antalpa1) in Antalpa1-treated animals. Collagen I (COL-I)/GFP co-positive cells also decreased from 85% in the control (Fig. 4B and E, Control) to less than 60% in the treated mice (Fig. 4B and E, Antalpa1). Based on these observations, it is likely that inhibition of LPA-LPA1 signalling does not affect bone marrow-derived cell extravasation, but prevents its differentiation into myofibroblasts.

It has been reported that bone marrow-derived fibrocytes are one of the cell sources of myofibroblasts 24. It showed that CD45 and COL-I were positively expressed in fibrocytes 25–29. To explore whether Antalpa1 selectively inhibits BMSC differentiation into myofibroblasts or it can also inhibit fibrocyte differentiation into myofibroblasts, we co-stained fibrocyte markers-CD45 and COL-I with the myofibrocyte marker-α-SMA in lung sections. As shown in Figure S2C, there was no significant change in the proportion of CD45/COL-I/α-SMA cells between these two groups. These data suggest that inhibition of LPA-LPA1 signal pathway selectively prevents BMSC differentiation into myofibroblasts in the fibrotic lung.

**Figure 5:**
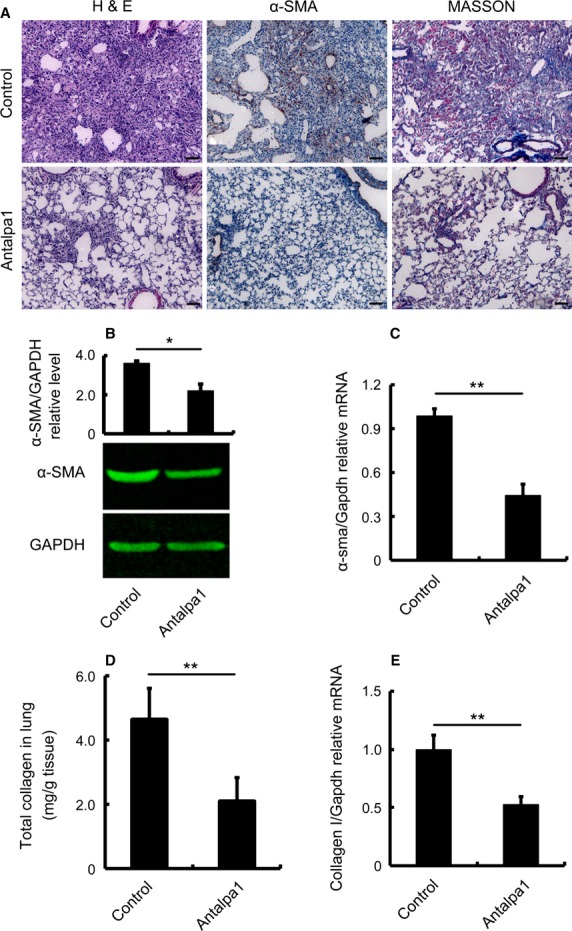
Antalpa1 markedly attenuates lung fibrosis induced by bleomycin. (A) Typical photomicrographs of haematoxylin and eosin, immunohistochemical staining for α-SMA, and Masson Trichrome staining of the lung from bleomycin-(BLM) challenged mice with Control (upper) or Antalpa1 (lower) injection. (B) Protein levels of α-SMA in the lung tissue of the above two groups detected by western blot analysis. (C) Relative mRNA expression of *α-sma* in the lung tissue of the above two groups detected by quantitative RT-PCR analysis. (D) Total protein levels of collagen in the lung tissue of the above two groups detected by Sircol collagen assay. (E) Relative mRNA expression of collagen I in the lung tissue of the above two groups detected by quantitative RT-PCR analysis. Each value represents the mean ± SE. *n* = 6 mice in each group. **P < 0.01; *P < 0.05.

To test the effect of Antapla1 on lung fibrosis in mice challenged with bleomycin, we measured the production of α-SMA as well as collagens in the damaged lung tissues. Indeed, Antalpa1 treatment reduced the production of both α-SMA (Fig. 5A and B, Antalpa1) and collagen (Fig. 5A and D, Antalpa1), and these effects most likely occurred through transcriptional regulation, as the *α-sma* mRNA (Fig. 5C, Antalpa1) as well as collagen I mRNA (Fig. 5E, Antalpa1) both decreased significantly. Based on these biomarkers, we conclude that inhibition of LPA-LPA1 signalling attenuates lung fibrosis in BLM-induced animals.

### Human BMSC involvement in lung injury induced by bleomycin in SCID/Beige mice

**Figure 6:**
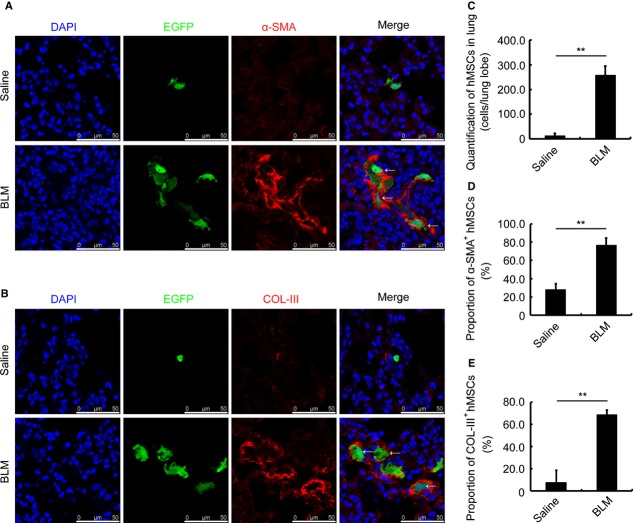
hBMSCs are involved in lung injury of SCID/Beige mice. (A) Immunofluorescence staining for α-SMA (red) in the lung of Saline- (upper) or BLM-(lower) challenged SCID/Beige mice. The mice were transplanted with GFP-labelled hBMSCs 48 hrs after bleomycin administration. DAPI (blue) staining shows the nucleus. α-SMA-positive cells are indicated with arrows. (B) Immunofluorescence staining for collagen III (COL-III, red) in the lung of Saline- (upper) or BLM-(lower) challenged SCID/Beige mice. The mice were transplanted with GFP-labelled hBMSCs 48 hrs after bleomycin administration. DAPI (blue) staining shows the nucleus. The COL-III-positive cells are indicated with arrows. (C) Quantification of accumulated hBMSCs in the lung of Saline- and BLM-treated mice. (D) Proportion of α-SMA-positive hBMSCs in the lung of Saline- and BLM-treated mice. (E) Proportion of COL-III-positive hBMSCs in the lung of Saline- and BLM-treated mice. All the lung tissues were isolated at day 7 after saline or BLM administration. Each value represents the mean ± SE. *n* = 6 mice in each group. **P < 0.01. BLM: bleomycin.

To further prove the effect of MSCs on lung injury, we transplanted GFP-labelled hBMSCs into SCID/Beige mice challenged with bleomycin. We found that bleomycin could effectively induce lung injury in SCID/Beige mice (Fig. S3D). Human BMSCs accumulated significantly in the injured lung, and the total number of hBMSCs increased most markedly in day 4 after bleomycin administration (Fig. S3A and B, day4). The accumulation of hBMSCs lasted up to day 14 although the total number decreased than that at day 4 (Fig. S3A and B, day14). We then determined the composition of transplanted hBMSCs in the injured lung. We found that compared to that in the lung of saline-treated mice, nearly 80% of GFP-positive hBMSCs expressed α-SMA in the BLM group at 7 days (Fig. 6A and D, BLM) or at 14 days (Fig. S3A, day14) after BLM administration. Furthermore, almost 70% of the accumulated hBMSC in the injured lung secreted collagen III at day 7 of bleomycin challenge (Fig. 6B and E, BLM). Additionally, most of the migrated hBMSC secreted collagen I 14 days after BLM administration (Fig. S3C, BLM). From the above data, we document that the transplanted hBMSCs are involved in lung injury, regardless of early or late timing after BLM challenge.

### Antalpa1 attenuates lung injury by inhibiting hBMSC differentiation in SCID/Beige mice

**Figure 7:**
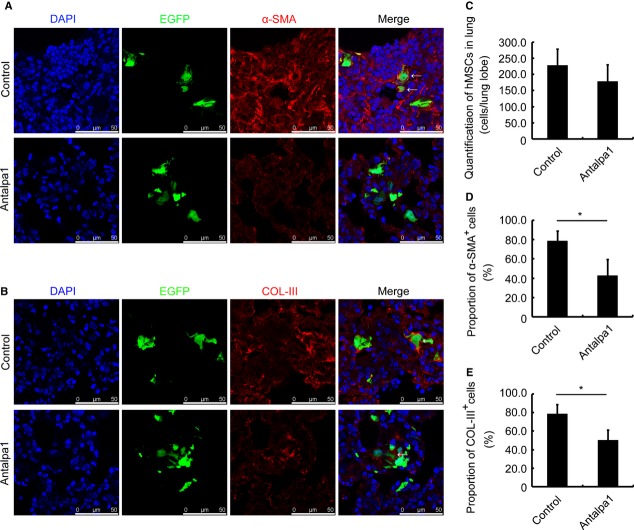
Antalpa1 reduces hBMSC differentiation into myofibroblasts and the secretion of extracellular matrix in the bleomycin-injured lung. (A) Immunofluorescence staining for α-SMA (red) in the lung of bleomycin-challenged SCID/Beige mice with Control (upper) or Antalpa1 (lower) injection. Mice were transplanted with GFP-labelled hBMSCs 48 hrs after bleomycin administration. DAPI (blue) staining shows the nucleus. α-SMA-positive cells are indicated with arrows. (B) Immunofluorescence staining for COL-III (red) in the lung of bleomycin-challenged SCID/Beige mice with Control (upper) or Antalpa1 (lower) injection. The mice were transplanted with GFP-labelled hBMSCs 48 hrs after bleomycin administration. DAPI (blue) staining shows the nucleus. The COL-III positive cells are indicated with arrows. (C) Quantification of accumulated hBMSCs in the lung of bleomycin-challenged SCID/Beige mice with Control or Antalpa1 injection. (D) Proportion of α-SMA-positive hBMSCs in the lung of bleomycin-challenged SCID/Beige mice with Control or Antalpa1 injection. (E) Proportion of COL-III-positive hBMSCs in the lung of bleomycin-challenged SCID/Beige mice with Control or Antalpa1 injection. All lung tissues were isolated at day 7 after saline or BLM administration. Each value represents the mean ± SE. *n* = 6 mice in each group. **P < 0.01. BLM: bleomycin.

To further test the effect of Antalpa1 on SCID/Beige mice with GFP-positive hBMSC transplantation after bleomycin administration, we detected the proportion of myofibroblasts derived from hBMSCs and ECM secretion by hBMSCs in mice treated with Antalpa1 for 7 days. We found that the proportion of α-SMA-positive hBMSCs decreased from 80% (Fig. 7A and D, Control) to 40% in Antalpa1-treated animals (Fig. 7A and D, Antalpa1). As well, the proportion of collagen III (COL-III)-positive hBMSCs decreased from nearly 80% (Fig. 7B and E, Control) to 50% in Antalpa1-treated mice (Fig. 7B and E, Antalpa1). However, as compared to that in the lung of control mice, the total number of accumulated hBMSCs remained unchanged in Antalpa1-treated mice (Fig. 7 A–C). In addition, this was also the case for mice treated with Antalpa1 for 14 days (data not shown). Based on these observations, consistent with Figures 3 and 4 and Fig. S2A and B, it is likely that inhibition of LPA-LPA1 signalling does not affect hBMSC extravasation, but prevents differentiation of hBMSCs into myofibroblasts along with the secretion of ECM. Furthermore, Antalpa1 attenuated the injury induced by BLM in SCID/Beige mice lung significantly either 7 or 14 days after injection (data not shown).

## Discussion

Pulmonary fibrosis is a common pathological process and more than 100 clinical diseases can induce these disorders 45. For example, idiopathic pulmonary fibrosis (IPF), the most common and serious lung interstitial disease, can be triggered by allergens, toxic chemicals, radiation, other persistent irritants and unknown factors. In the European Union, the prevalence of IPF is 26 per 100,000 annually and the patients have a median survival time of only 2–3 years after diagnosis 46. However, there is no satisfying therapy to treat pulmonary fibrosis presently. The frequently used corticosteroids and immunosuppressive drugs have too many side effects. Therefore, it is necessary to find satisfactory and effective new drugs through determination of the underlying mechanism of pulmonary fibrosis.

Myofibroblast, the major cell type contributes to pulmonary fibrosis, activates and expresses α-SMA under the stimulation of pro-fibrogenic factors 2. However, the cellular source of myofibroblast is contradictory. It has been reported that myofibroblasts could be derived from resident fibroblasts 5, epithelial–mesenchymal transition 9, and fibrocytes 24 in the fibrotic lung. However, in our present study, we observed that the proportion of BMSC-derived myofibroblasts increased nearly 30 times, and about 40% of α-SMA-positive cells were derived from BMSCs in the mouse lung after BLM treatment. We therefore concluded that BMSCs are one of the major cell sources of myofibroblasts in the fibrotic lung. We further provided mechanism of differentiation of BMSCs into myofibroblasts that is mainly responsible for fibrosis in BLM-treated mouse lung. By targeting at the LPA-LPA1 signalling, a novel LPA1 antagonist-Antapla1 could attenuate fibrotic conditions in BLM-treated mouse lung.

To our knowledge, this is the first study to propose that differentiation of BMSCs to myofiboblasts mediated by LPA-LPA1 is a major mechanism for pulmonary fibrosis. Nonetheless, we realized some limitations for our conclusion. First, all our data were from BLM-treated mice model of pulmonary fibrosis. We are not sure whether this is also applicable to other pulmonary fibrosis models, not to mention the human case. Second, we noted that about 40% of α-SMA-positive cells were derived from BMSCs (Fig. S4A, BLM) in the mouse lung after BLM treatment. However, CD44/Sca-1/α-SMA triple positive cells occupy only one third of bone marrow-derived ‘green cells’ (Fig. 1B and F, BLM). These suggest that some other cells in either lung or bone marrow might also become myofibroblasts during pulmonary fibrosis. Nonetheless, bone marrow-derived MSCs are indeed one of the major cell sources for myofibroblasts.

Conversely, many studies have reported that MSC transplantation could treat a number of diseases. Some scientists even suggest MSC transplantation could be used in clinical trials. However, based on our either endogenous or exogenous transplantation of MSCs to BLM-challenged mice, we observed that BMSCs accumulated significantly in the damaged lung. Furthermore, most of the transplanted hBMSCs differentiated into myofibroblasts, and secreted ECM in early (7 days) and late (14 days) time-points after BLM administration (Fig. 6 and Fig. S3A–C). In fact, we also transplanted EGFP transgenic mBMSCs (Fig. S4D) into the BLM-challenged SCID/Beige mice and found that most of the transplanted mBMSCs also differentiated into myofibroblasts and secreted ECM (Fig. S5A and B) in the fibrotic lung. Considering that BMSCs might contribute to fibrotic diseases, caution must be exercised in the clinical use of MSC therapies and it might be much safer to transplant the MSCs after committed differentiation into certain functional cells.

Lysophosphatidic acid, a multi-functional phospholipid, regulates many physiopathological activities *via* its six receptors (LPA1–6) 32. It has been reported that LPA-LPA1 signalling contributes to lung fibrosis by regulating vascular leakage and recruitment of fibroblasts 3–4. Tager *et al*. proposed that such regulation by LPA-LPA1 was through the recruitment of resident fibroblasts, but not through the recruitment of circulating bone marrow-derived cells 4. In this study, we provide further mechanism of LPA-LPA1 signalling-mediated differentiation of BMSCs into myofibroblasts during lung fibrosis. We also showed that a novel LPA1-specific inhibitor, Antapla1, did not affect the extravasation of bone marrow-derived cells (Fig. 4A and B, EGFP and Fig. 4C; Fig. 7A and B, EGFP and Fig. 7C). Our observation is in agreement with that of Tager *et al*. However, unlike in their report, we found that nearly half of the myofibroblasts in the damaged lung were actually from BMSCs.

It has been reported that pulmonary fibrosis could also be attenuated by inhibiting fibrocyte differentiation into myofibroblasts 13 or inhibiting lung epithelial cell apoptosis 47. However, we did not find any significant change in the proportion of fibrocyte-derived myofibroblasts between the Antalpa1-treated and control group mice (Fig. S2C). For further detection the effect of Antalpa1 on lung epithelial cell apoptosis, we detected the proportion of Annexin V/PI-positive cells 72 hrs after LPA stimulation with or without Antalpa1 pre-treatment in human bronchial epithelial cell (16HBE). Consistent with a previous study 47, we found that LPA (10 μM) could indeed induce the apoptosis of 16HBE and the proportion of Annexin V/PI-positive cells returned to the control level when the cells were pre-treated with 30 μM Antalpa1 (Fig. S6A). These results indicate that in addition to blocking the differentiation of MSCs into myofibroblasts, the effect of Antalpa1 attenuation lung fibrosis might also be mediated by inhibiting the apoptosis of lung epithelial cells induced by LPA. All these imply that lung fibrosis is a complicated process and more work is needed to clarify the mechanism of Antalpa1 on lung cell apoptosis *in vivo*.

Recently, many studies have proved that the abnormal increased expression level of LPA in bronchoalveolar was relevant to IPF. In this study, we proved that Antalpa1 could attenuate bleomycin-induced pulmonary fibrosis effectively. However, we do not have an answer as to whether Antalpa1 could also be used to treat other pulmonary fibrosis such as IPF. Furthermore, increasingly more potent and specific LPA1 antagonists have been identified and entered in phase I clinical trials 3–48. Antalpa1, a novel chemical LPA1 antagonist used in this study, represents an additional clinical candidate. It would be interesting to compare the efficacy of these compounds in animal models and human clinical trials in the treatment of fibrosis of various tissues. More work is needed for us in future to explore the potential clinical value of Antalpa1.

It has been reported that the level of LPA was elevated several times under certain pathological conditions such as ovary cancer 37–40. All these may be involved in LPA-mediated phosphorylation of ERK and Akt 49–50. Consistent with these reports, we found that the phosphorylation level of ERK and Akt increased significantly at 30 and 60 min. after LPA stimulation in hBMSCs (Fig. S6B, upper panel). Furthermore, Ki16425 and Antalpa1 could totally abrogate the phosphorylation of ERK. However, unlike Ki16425, Antalpa1 could not inhibit the activation of Akt effectively (Fig. S6B, lower panel). These data suggest that ERK activation is mediated *via* LPA1, and the differentiation of hBMSC into myofibroblasts induced by LPA might depend on ERK activation.

Meanwhile, there is another possibility that the migration abilities of BMSCs derived from mice with or without BLM challenge are different. To further test the migration ability of BMSCs isolated at different time-points (day 3, 7 and 14) in BLM-treated mice, we performed the Chamber Boyden Assay. We found that compared to that in saline-challenged mice, the migration significantly increased in BMSCs isolated from BLM-treated mice than that in saline controls. This was observed in the cells isolated at days 3 and 7 (Fig. S6C and D), but not at day 14 (Fig. S6E). This might result from the fact that LPA concentration has reached the basal level at day 14 in the lung (Fig. 2F). From these phenomena, we propose that BMSCs are more able to migrate in the early or middle stage of lung injury. The underlying mechanism needs to be investigated in future.

## Conclusion

Our study not only elucidates a novel LPA-LPA1 pathway that induces BMSC differentiation into myofibroblasts thereby contributing to lung fibrosis but also provides more evidence for LPA1 as a potential therapeutic target for fibrosis disease treatment.

## Supporting information

Figure S1 Bleomycin markedly induces lung fibrosis and BMSCs accumulated significantly in the fibrotic lung.

Figure S2 Antalpa1 barely inhibits LPA-induced hBMSC migration *in vitro* and Antalpa1 selectively inhibit BMSC differentiation into myofibroblast *in vivo*.

Figure S3 GFP-labelled hBMSCs in the injured lung of SCID/Beige mice.

Figure S4 Statistical Analysis of proportions of different cell sourcederived myofibroblasts and characterization of mBMSCs by flow cytometry.

Figure S5 Purified EGFP positive mBMSCs involved in lung fibrosis.

Figure S6 The Multifunction of LPA to various cells.

Table S1 Information of used antibodies.

Table S2 Sequences of primers for Q-PCR.

Table S3 Inhibition of LPA-induced activities by Antalpa1, IC_50_ (_μ_M).

Data S1 Production information of Antalpa1 (CGX1002).
