# Supplementary figures and images for: Lysophosphatidic acid accelerates lung fibrosis by inducing differentiation of mesenchymal stem cells into myofibroblasts
Source: J Cell Mol Med. 2013 Nov 19;18(1):156–69. doi: 10.1111/jcmm.12178 (PMC3916127; doi:10.1111/jcmm.12178)

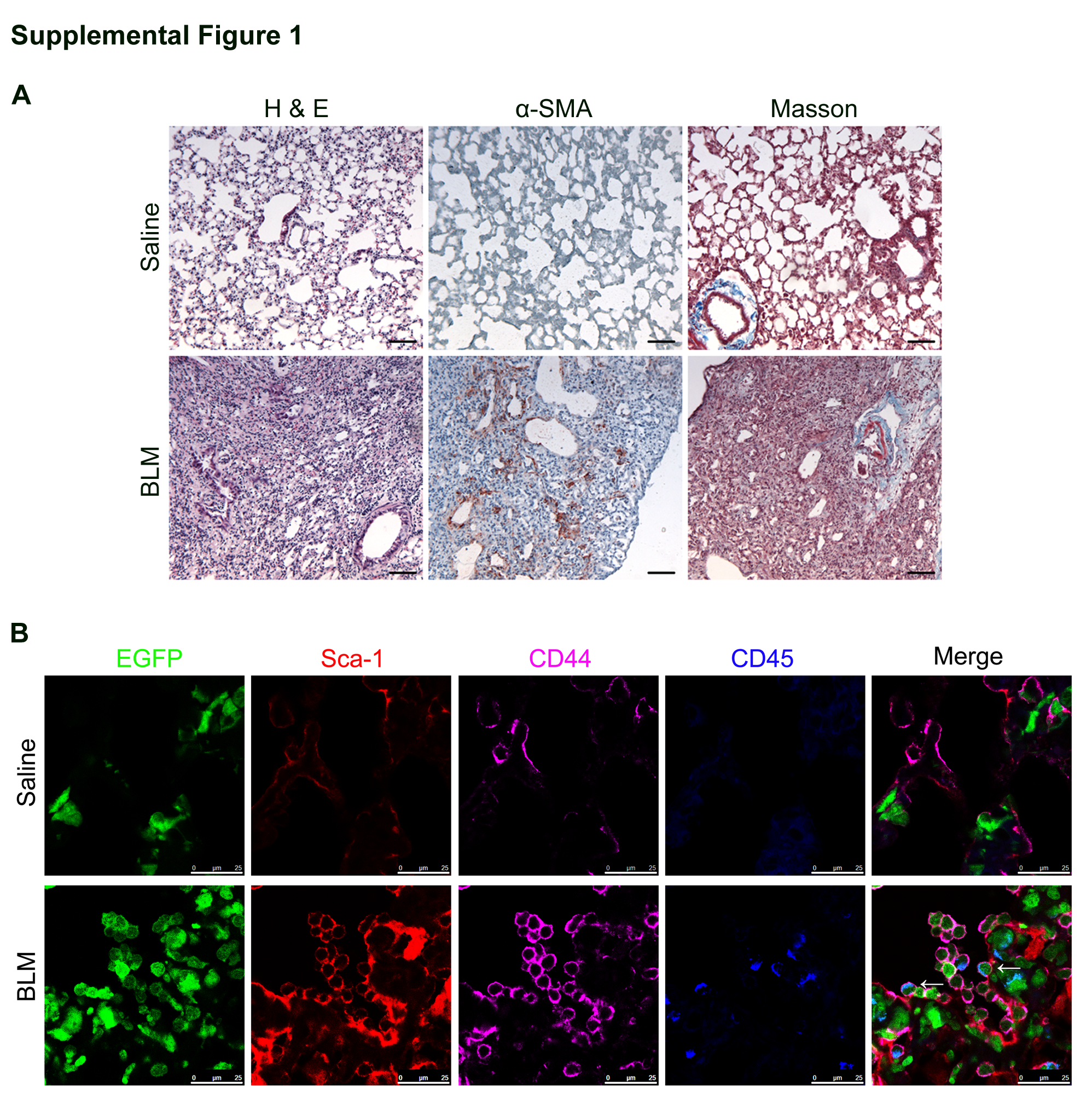

Supplement: Figure S1 — Bleomycin markedly induces lung fibrosis and BMSCs accumulated significantly in the fibrotic lung. [file jcmm0018-0156-sd1.tif]

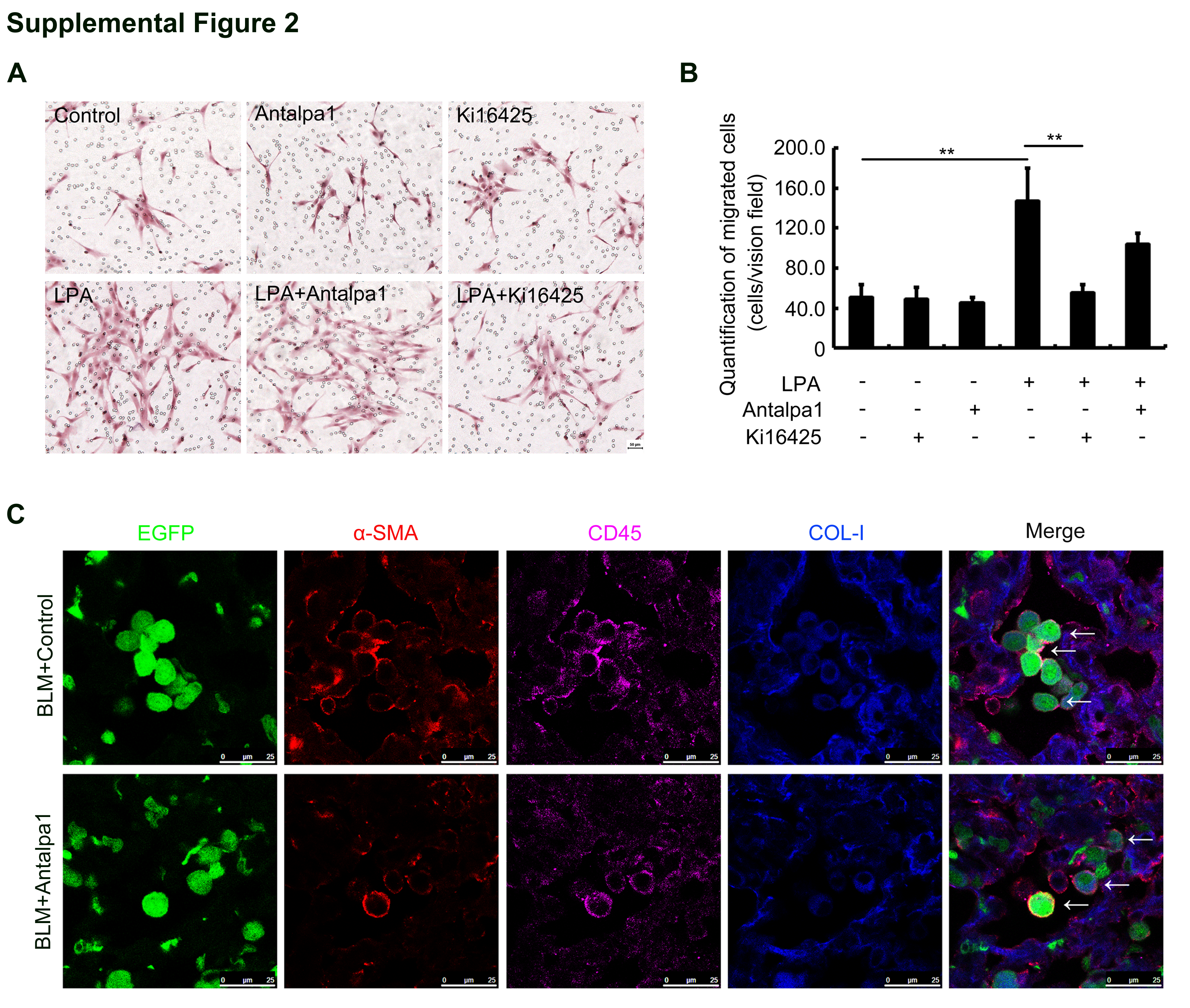

Supplement: Figure S2 — Antalpa1 barely inhibits LPA-induced hBMSC migration in vitro and Antalpa1 selectively inhibit BMSC differentiation into myofibroblast in vivo. [file jcmm0018-0156-sd2.tif]

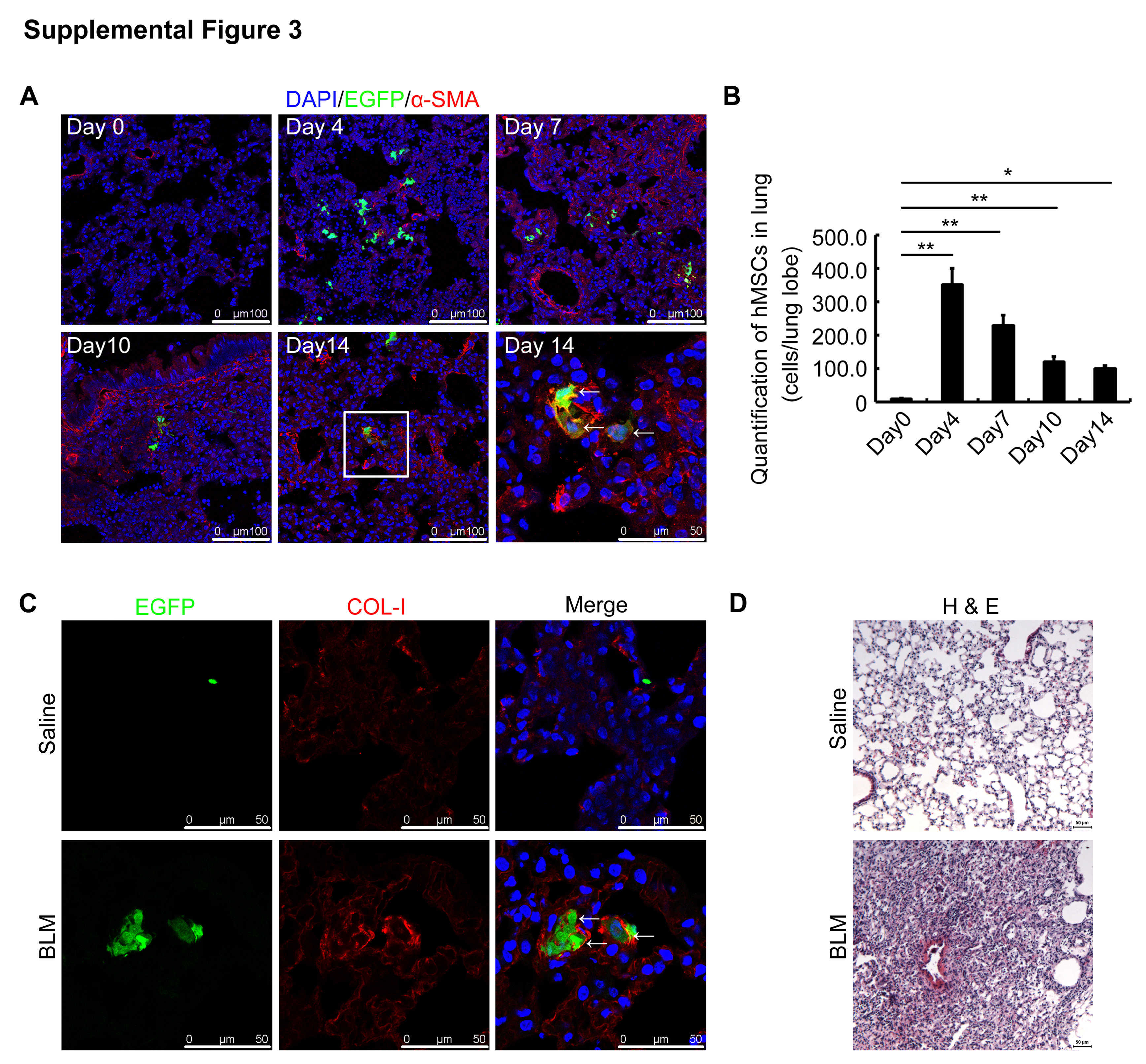

Supplement: Figure S3 — GFP-labelled hBMSCs in the injured lung of SCID/Beige mice. [file jcmm0018-0156-sd3.tif]

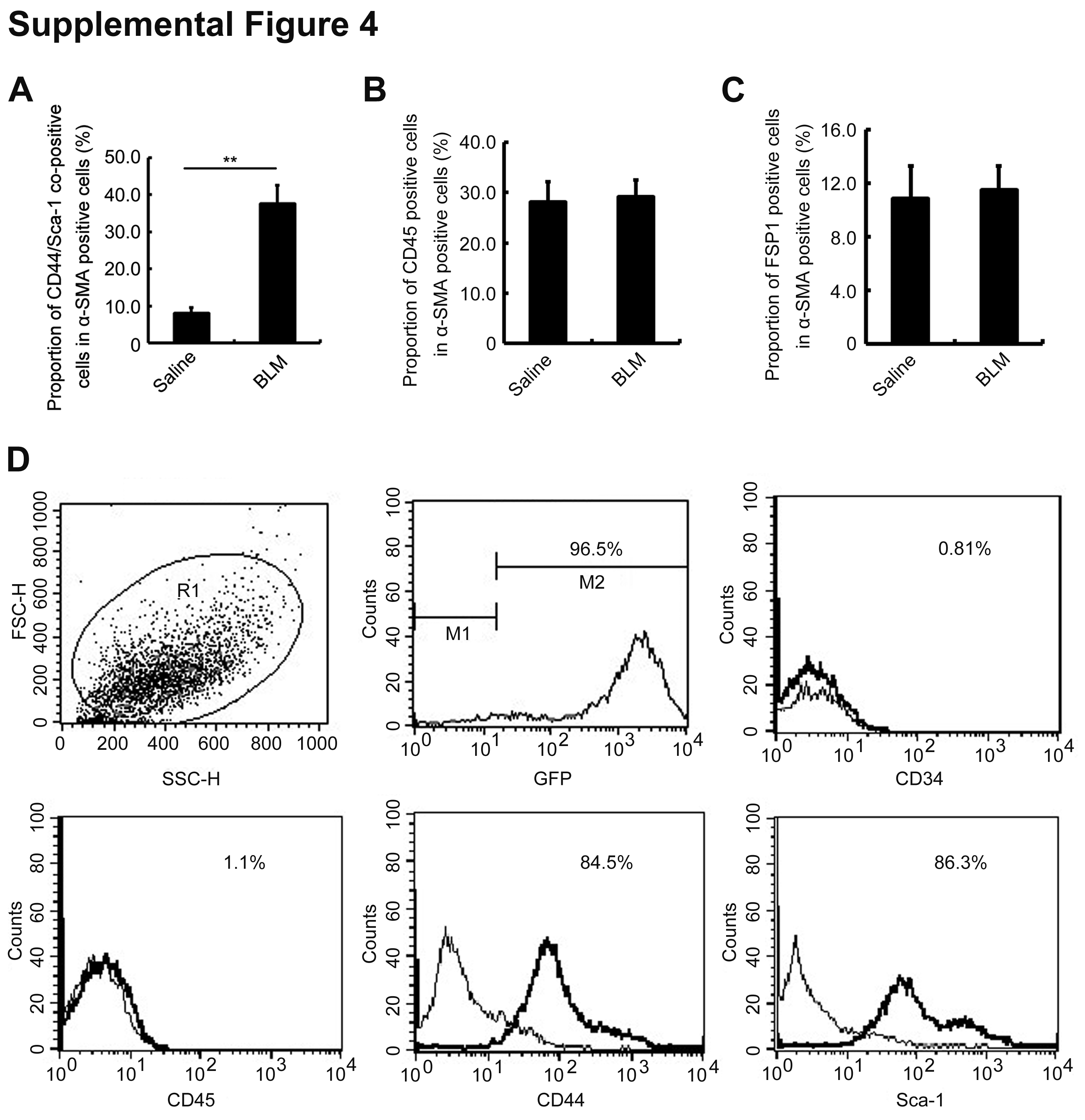

Supplement: Figure S4 — Statistical Analysis of proportions of different cell sourcederived myofibroblasts and characterization of mBMSCs by flow cytometry. [file jcmm0018-0156-sd4.tif]

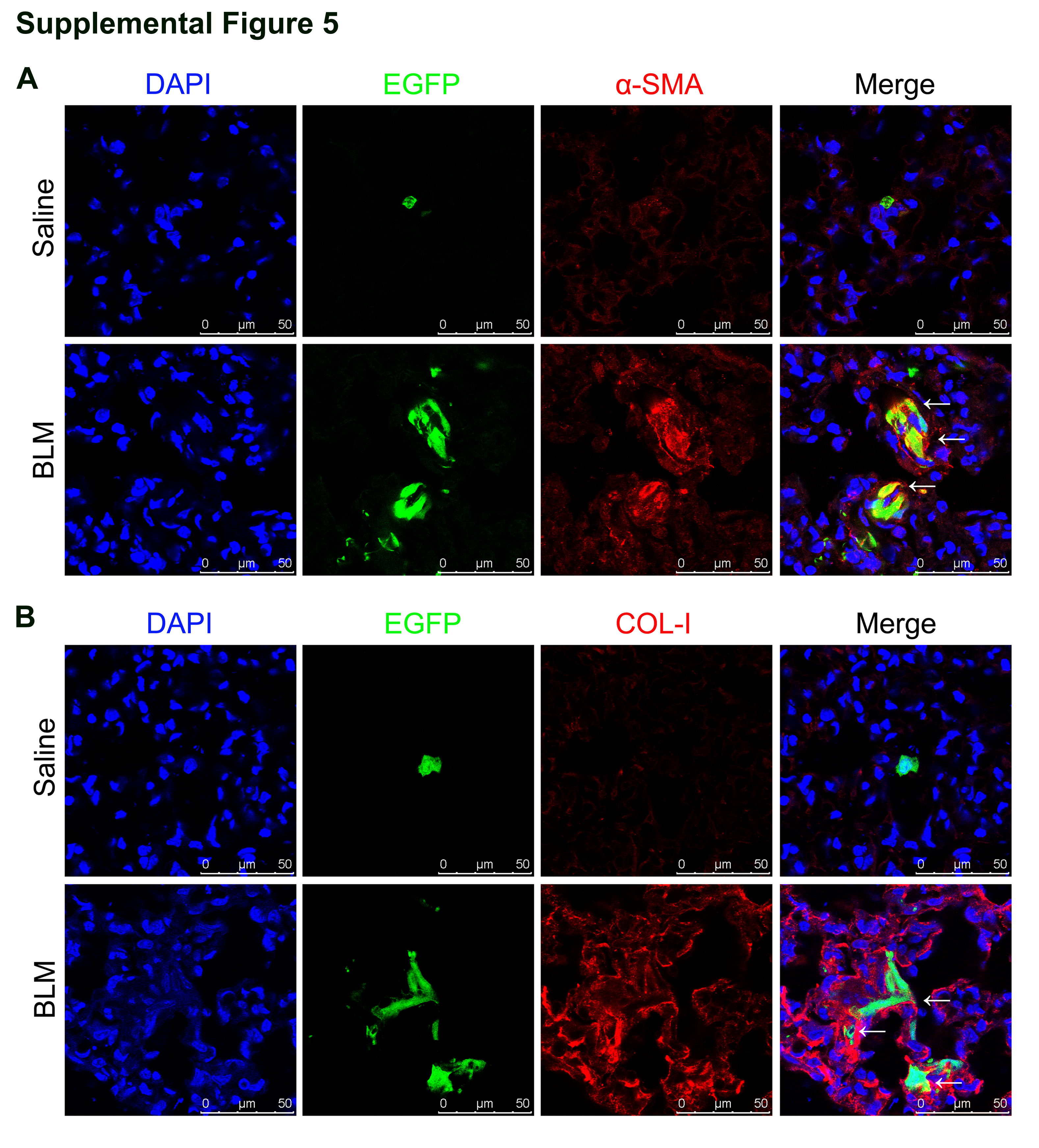

Supplement: Figure S5 — Purified EGFP positive mBMSCs involved in lung fibrosis. [file jcmm0018-0156-sd5.tif]

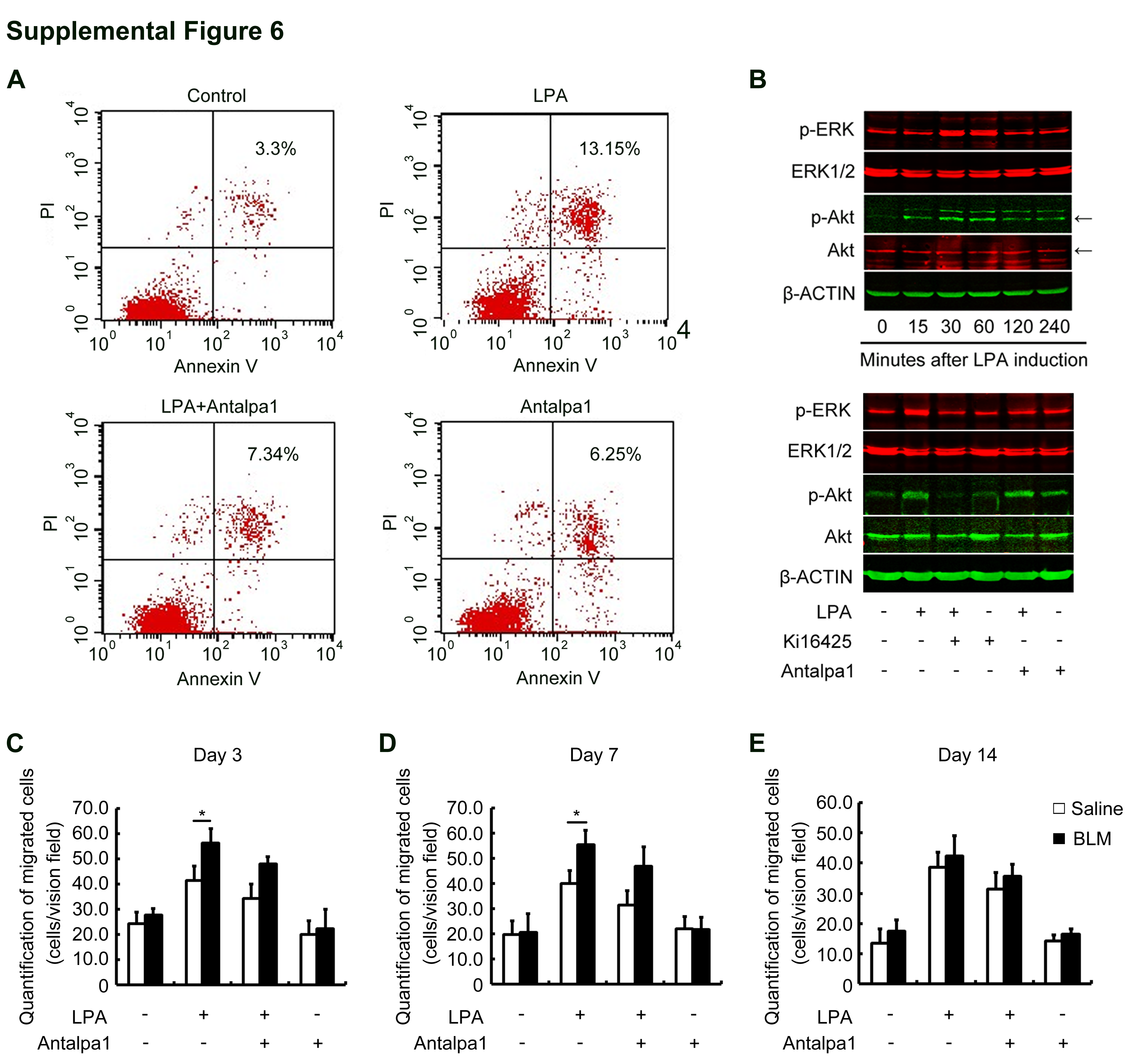

Supplement: Figure S6 — The Multifunction of LPA to various cells. [file jcmm0018-0156-sd6.tif]
