# Supplementary material for: Lysophosphatidic acid accelerates lung fibrosis by inducing differentiation of mesenchymal stem cells into myofibroblasts
Source: J Cell Mol Med. 2013 Nov 19;18(1):156–69. doi: 10.1111/jcmm.12178 (PMC3916127; doi:10.1111/jcmm.12178)
Supplement: Table S1 — Information of used antibodies. [file jcmm0018-0156-sd7.docx]

**Supplementary Table 1:** Information of used antibodies

| **Antibody** | **Source** | **Company** | **Dilution** |
| --- | --- | --- | --- |
| α-SMA | Mouse monoclonal | sigma | 1:1000 |
| α-SMA | Rabbit polyclonal | abcam | 1:100 |
| collagen type I | Mouse monoclonal | sigma | 1:500 |
| collagen type I | Rabbit polyclonal | Millipore | 1:100 |
| collagen type III | Rabbit polyclonal | abcam | 1:100 |
| CD44 | Mouse monoclonal | Cell Signaling Technologies | 1:1000 |
| CD44 | Rat polyclonal | abcam | 1:100 |
| CD45 | Rat polyclonal | abcam | 1:50 |
| CD45 | Rabbit polyclonal | abcam | 1:50 |
| Sca-1 | Rat polyclonal | abcam | 1:50 |
| ERK1/2 | Rabbit polyclonal | Santa Cruz | 1:1000 |
| p-ERK | Rabbit polyclonal | Santa Cruz | 1:1000 |
| Akt | Rabbit polyclonal | Cell Signaling Technologies | 1:1000 |
| p-Akt | Mouse monoclonal | Cell Signaling Technologies | 1:1000 |
| β-ACTIN | Mouse monoclonal | Sigma | 1:10000 |
| GAPDH | Mouse monoclonal | ZhongShan | 1:2000 |
| Alexa Fluor 594 | Goat anti- rabbit IgG | ZhongShan | 1:400 |
| Alexa Fluor 594 | Goat anti-mouse IgG | ZhongShan | 1:400 |
| Alexa Fluor 488 | Goat anti-mouse IgG | ZhongShan | 1:400 |
| Alexa Fluor 488 | Goat anti-rabbit | ZhongShan | 1:400 |
| IgG-CFL 647 | Goat anti-rat | Santa Cruz | 1:50 |
| IgG-CFL 405 | Goat anti-rabbit | Santa Cruz | 1:50 |
| **Definition of abbreviation:**  α-SMA: α-smooth muscle actin. | | | |
