# Supplementary material for: Lysophosphatidic acid accelerates lung fibrosis by inducing differentiation of mesenchymal stem cells into myofibroblasts
Source: J Cell Mol Med. 2013 Nov 19;18(1):156–69. doi: 10.1111/jcmm.12178 (PMC3916127; doi:10.1111/jcmm.12178)
Supplement: Table S2 — Sequences of primers for Q-PCR. [file jcmm0018-0156-sd8.docx]

**Supplementary Table 2: Sequences of primers for Q-PCR**

| **genes** | **primer sequences** |
| --- | --- |
| Mus α-sma | F: 5’-ATGCTCCCAGGGCTGTTTT-3’ |
|  | R: 5’-TTCCAACCATTACTCCCTGATGT-3’ |
| Mus Collagen I | F: 5’-AGGGCGAGTGCTGTGCTTT-3’ |
|  | R: 5’-CCCTCGACTCCTACATCTTCTGA-3’ |
| Mus Gapdh | F: 5’-TCCCACTCTTCCACCTTCGATGC-3’ |
|  | R: 5’-GGGTCTGGGATGGAAATTGTGAGG-3’ |
| Homo α-sma | F: 5'-GGTGATGGTGGGAATGGG-3' |
|  | R: 5'-GCAGGGTGGGATGCTCTT-3' |
| Homo Collagen I | F: 5'-CTGGTCTTCCTGGCCCCTCTGGTG-3' |
|  | R: 5'-CCTCTGTCGCCCTGTTCGCCTGTC-3' |
| Homo Gapdh | F: 5'-CATGAGAAGTATGACAACAGCCT-3' |
|  | R: 5'-AGTCCTTCCACGATACCAAAGT-3' |
| Definition of abbreviations:  Mus: mouse; Homo: human; α-sma: alpha-smooth muscle actin; Gapdh: glyceraldehydes-3-phosphate dehydrogenase | |
