# Supplementary material for: Lysophosphatidic acid accelerates lung fibrosis by inducing differentiation of mesenchymal stem cells into myofibroblasts
Source: J Cell Mol Med. 2013 Nov 19;18(1):156–69. doi: 10.1111/jcmm.12178 (PMC3916127; doi:10.1111/jcmm.12178)
Supplement: Table S3 — Inhibition of LPA-induced activities by Antalpa1, IC50 (μM). [file jcmm0018-0156-sd9.docx]

**Supplementary Table 3: Inhibition of LPA-induced activities by Antalpa1, IC50 (uM)**

|  | **LPA1** | **LPA2** | **LPA3** |
| --- | --- | --- | --- |
| SRE reporter gene assay | 0.25 | >30 | >30 |
| Ca2+ influx assay | 0.09 | >30 | >30 |
