# Supplementary material for: Lysophosphatidic acid accelerates lung fibrosis by inducing differentiation of mesenchymal stem cells into myofibroblasts
Source: J Cell Mol Med. 2013 Nov 19;18(1):156–69. doi: 10.1111/jcmm.12178 (PMC3916127; doi:10.1111/jcmm.12178)
Supplement: Data S1 — Production information of Antalpa1 (CGX1002). [file jcmm0018-0156-sd10.docx]

**Supplementary Figure Legends**

**Supplementary Figure 1.** Bleomycin markedly induces lung fibrosis and BMSCs accumulated significantly in the fibrotic lung. **(A):** Typical photomicrographs of H & E, immunohistochemisical staining for α-SMA, and Masson Trichrome staining in the lung of mice 14 days after intratracheal administration of Saline (upper) or BLM (lower). **(B):** Immunofluorescence staining of Sca-1 (red), CD44 (purple) and CD45 (blue) in the lung sections of EGFP bone marrow replaced mice 14 days after Saline or BLM administration. The Sca-1/CD44/CD45 triple positive “green” cells are indicated with arrows. BLM: bleomycin.

**Supplementary Figure 2.** Antalpa1 barely inhibits LPA-induced hBMSC migration in vitro and Antalpa1 selectively inhibit BMSC differentiation into myofibroblast in vivo. **(A):** Typical photomicrographs of H & E staining of the lower surface of Boyden chambers filter. Images show the migrated hBMSC without LPA addition (Control), Antalpa1 (30μM), Ki16425 (30μM), LPA (10μM), LPA (10μM) with Antalpa1 (30μM) pretreatment (LPA + Antalpa1) and LPA (10μM) with Ki16425 (30μM) pretreatment (LPA + Ki16425). **(B):** Quantification of migrated hBMSC in the above groups. For the quantification of migrated cells, 15 visual fields were taken randomly in each group. Each value represents the mean ± SE. ***P* <0.01. The experiment was repeated more than 3 times. **(C):** Immunofluorescence staining of α-SMA (red), CD45 (purple) and COL-I (blue) in the lung of EGFP bone marrow replaced mice 14 days after Saline or BLM administration. The mice were treated with or without Antalpa1 for 14 days. The α-SMA /CD45/COL-I triple positive “green” cells are indicated with arrows. BLM: bleomycin.

**Supplementary Figure 3.** GFP labeled hBMSCs in the injured lung of SCID/Beige mice. **(A):** Immunofluorescence staining for α-SMA (red) in the lung of bleomycin challenged SCID/Beige mice with GFP-labeled hBMSC transplantation. DAPI (blue) staining shows the nucleus. The α-SMA positive cells are indicated with arrows**. (B):** Quantification of hBMSCs in the lung of SCID/Beige mice 0, 4, 7, 10, and 14 days after bleomycin administration. (C): Immunofluorescence staining for COL-I (red) in the lung of bleomycin challenged SCID/Beige mice with GFP-labeled hBMSC transplantation. Lung tissues were isolated in Day 14 after bleomycin administration. DAPI (blue) staining shows the nucleus. The COL-I positive cells are indicated with arrows**.** (D) Typical photomicrographs of H & E staining of the lung tissues from SCID/Beige mice 14 days after Saline and BLM administration. Each value represents the mean ± SE. ***P* <0.01; **P* <0.05. n = 6 mice in each group. BLM: bleomycin.

**Supplementary Figure 4.** Statistical Analysis of proportions of different cell source-derived myofibroblasts and characterization of mBMSCs by flow cytometry. **(A)** Quantifiction of CD44/Sca-1 co-positive “green” cells in α-SMA positive cells. **(B)** Quantifiction of CD45 positive “green” cells in α-SMA positive cells. **(C)** Quantifiction of FSP1 positive “green” cells in α-SMA positive cells. Each value represents the mean ± SE. ***P* <0.01; **P* <0.05. n = 6 mice in each group. BLM: bleomycin. **(D)** Characterization of EGFP transgenic mBMSCs by flow cytometry.

**Supplementary Figure 5.** Purified EGFP positive mBMSCs involved in lung fibrosis. **(A):** Immunofluorescence staining for α-SMA (red) in the lung of Saline (upper) or BLM (lower) challenged SCID/Beige mice. The mice were transplanted with EGFP positive mBMSCs 48 hours after bleomycin administration. DAPI (blue) staining shows the nucleus. α-SMA-positive “green” cells are indicated with arrows. **(B):** Immunofluorescence staining for collagen I (COL-I, red) in the lung of Saline (upper) or BLM (lower) challenged SCID/Beige mice. The mice were transplanted with EGFP positive mBMSCs 48 hours after bleomycin administration. DAPI (blue) staining shows the nucleus. The COL-I-positive cells are indicated with arrows.

**Supplementary Figure 6.** The Multifunction of LPA to various cells. **(A)** The apoptotic cells were detected by Annexin V/PI staining. The cells were pre-treated with 30μM Antalpa1 before addition of 10μM of LPA. **(B):** Protein levels of ERK1/2, p-ERK, Akt and p-Akt in hBMSCs 0, 15, 30, 60 and 120 minutes after LPA induction were detected by western blot analysis (upper panel). Protein levels of ERK1/2, p-ERK, Akt and p-Akt in hBMSCs 30 minutes after LPA induction were detected by western blot analysis (lower panel). The cells were pretreated with or without Antalpa1. **(C):** Migration abilities of mBMSCs derived from mice 3 days after saline or bleomycin administration. **(D):** Migration abilities of mBMSCs derived from mice 7 days after saline or bleomycin administration. **(E):** Migration abilities of mBMSCs derived from mice 14 days after saline or bleomycin administration. For the quantification of migrated cells, 15 visual fields were taken randomly in each group. Each value represents the mean ± SE. ***P* <0.01. The experiment was repeated more than 3 times.
