# Supplementary material for: Lysophosphatidic acid accelerates lung fibrosis by inducing differentiation of mesenchymal stem cells into myofibroblasts
Source: J Cell Mol Med. 2013 Nov 19;18(1):156–69. doi: 10.1111/jcmm.12178 (PMC3916127; doi:10.1111/jcmm.12178)
Supplement: Supplementary file 11 [file jcmm0018-0156-sd11.doc]

**Supplementary information for chemical synthesis of CGX-1002:**

**Step 1:**

Methyl acetoacetate (11.6g, 100mmol) was dissolved in 20mL MeOH. Methylamine (33 wt% in absolute ethanol, 18.7mL, 150mmol) was added into the solution very slowly at room temperature, and the reaction was stirred for 2 hours. The solvent was removed under the vacuum to give the final product (E)-methyl 3-(methylamino) but-2-enoate as white solid without further purification (12.9g).

**Step 2:**

(E)-methyl 3-(methylamino) but-2-enoate (12.9g, 100mmol) was dissolved in 300mL anhydrous THF and 12mL pyridine was added into the solution dropwise. The reaction was cooled in ice bath, and 4-nitrobenzoyl chloride (18.7mL in 50mL anhydrous THF) was added into the solution slowly. The reaction was warmed up to room temperature and stirred for overnight. After adding 300mL water into the reaction, the solution was extracted by ethyl acetate for 3 times. The combined organic layer was further washed with water and brine, dried over Na_2_SO_4_, and removed under the vacuum to give methyl 2-(4-nitrobenzoyl)-3-oxobutanoate as the solid without further purification (25.2g, 95% yield).

**Step 3:**

Methyl 2-(4-nitrobenzoyl)-3-oxobutanoate (20.0g, 75.47mmol) was dissolved in acetic acid (70mL), and followed by adding hydroxylamine hydrochloride (5.10g, 73.27mmol). The reaction was stirred at 115^o^C for 2 hours. After cooling the reaction to the RT, 400mL saturated NaHCO_3_ was added into the solution and extracted by ethyl acetate for 3 times. The combined organic layer was washed by brine and dried over Na_2_SO_4_, and removed under the vacuum. The crude product was further purified by flash chromatography using EA/Hexane=1:9 to give methyl 3-methyl-5-(4-nitrophenyl) isoxazole-4-carboxylate (16.4g, 83% yield). MS m/z 263.1 (M + 1)

**Step 4:**

Methyl 3-methyl-5-(4-nitrophenyl) isoxazole-4-carboxylate (13.1g, 50mmol) was dissolved in 150mL dioxane, and followed by adding 125mL lithium hydroxide (2N). The reaction was stirred at RT for overnight and neutralized by 6N HCl till pH<6. After removing the dioxane under the vacuum, the water solution was extracted by DCM for 3 times. The combined organic layer was washed by brine and dried over Na_2_SO_4_. After removing the solvent under the vacuum, the product 3-methyl-5-(4-nitrophenyl) isoxazole-4-carboxylic acid was used in next step reaction without further purification (10.66g, 86% yield). MS m/z 249.1 (M + 1)

Step 5:

3-methyl-5-(4-nitrophenyl)isoxazole-4-carboxylic acid (2.5g, 10mmol) was dissolved in 30mL toluene in a sealed tube, and followed by adding 1-(2-chlorophenyl)ethanol (1.88g, 12mmol), triethylamine (2.02g, 20mmol) and diphenylphosphoryl azide (4.13g, 15mmol). The reaction was stirred at 125^o^C for 2 hours. After cooling down the reaction to RT, the solvent was removed under the vacuum. The crude product was purified by flash chromatography using EA/Hexane (1:1) to get 1-(2-chlorophenyl) ethyl (3-methyl-5-(4-nitrophenyl) isoxazol-4-yl)carbamate (2.60g, 65% yield). MS m/z 402.1 (M + 1)

**Step 6:**

1-(2-chlorophenyl)ethyl (3-methyl-5-(4-nitrophenyl)isoxazol-4-yl) carbamate (2.60g, 6.48mmol) was dissolved in 100mL ethanol, followed by adding 260mg Pd/C (10% w/w). The reaction was stirred at RT under a hydrogen balloon for 4 hours. After filtering through a pad of celite, the solvent was removed under the vacuum to get the product 1-(2-chlorophenyl) ethyl (5-(4-aminophenyl)-3-methylisoxazol-4-yl) carbamate without further purification. MS m/z 372.1(M + 1)

**Step 7:**

1-(2-chlorophenyl)ethyl (5-(4-aminophenyl)-3-methylisoxazol-4-yl) carbamate (50mg, 0.13mmol) was dissolved in 5mL anhydrous DCM, followed by adding DIEA (47µL, 0.26mmol). Methanesulfonyl chloride (16.8mg, 0.15mmol) was added into the solution and the reaction was stirred at RT for 2 hours. After removing the solvent under the vacuum, the crude product was further purified by prep-TLC plate using 5%MeOH in DCM to get the final product 1-(2-chlorophenyl) ethyl (3-methyl-5-(4-(methylsulfonamido) phenyl) isoxazol-4-yl) carbamate (49mg, 82% yield). ^1^H NMR (400 MHz, CDCl_3_): δ1.28 (d, J=7.2Hz, 3H), 2.23 (s, 3H), 3.00 (s, 3H), 4.14 (q, J=7.2Hz, 1H), 5.32 (s, 1H), 7.13 (d, J=8.4Hz, 2H), 7.34-7.48 (m, 5H), 7.57 (d, J=7.6Hz, 1H), 9.02 (s, 1H), 9.61 (s, 1H) MS m/z 404.2 (M + 1). MS m/z 450.2 (M + 1)
